# Associations between hospital characteristics and the delivery of non-cancer palliative care: a nationwide retrospective study in Taiwan

**DOI:** 10.1186/s12904-026-02170-5

**Published:** 2026-06-01

**Authors:** Shen-Ju Tsai, Cheng-Pei Lin, Christy Pu, Wen-Hua Kuo

**Affiliations:** 1 Graduate Institute of Public Health, National Yang Ming Chiao Tung University, Taipei, 112304 Taiwan; 2 Department of Family Medicine, China Medical University Hospital, Taichung, 404328 Taiwan; 3 Department of Family Medicine, College of Medicine, China Medical University, Taichung, 404328 Taiwan; 4 Institute of Community Health Care, College of Nursing, National Yang Ming Chiao Tung University, Taipei, 112304 Taiwan; 5 Cicely Saunders Institute of Palliative Care, Policy and Rehabilitation, King’s College London, London, UK; 6 Department of Education and Research, Taipei City Hospital, Taipei, 203212 Taiwan

**Keywords:** Non-cancer palliative care, Hospital characteristics, Shared care model, Palliative care units, End-of-life care

## Abstract

**Background:**

As the population ages, the need for palliative care among patients with non-cancer conditions has increased. Although the benefits of palliative care for non-cancer conditions are well established, integration of such care remains limited across many health systems. Taiwan’s universal health system offers a unique opportunity to examine how hospital characteristics influence the delivery of non-cancer palliative care.

**Methods:**

We conducted a retrospective nationwide analysis of all hospitals providing palliative care through either dedicated palliative care units (PCUs) or palliative shared care (PSC) under Taiwan’s National Health Insurance (2016–2020). Non-cancer patients were identified based on the absence of cancer-related diagnostic codes within the National Health Insurance (NHI) claims data. The proportion of these patients receiving palliative care per hospital year was calculated. Generalized Estimating Equations (GEE) were used to stratify the data by admission year and hospital characteristics, and to assess the associations between these hospital characteristics and the delivery of non-cancer palliative care.

**Results:**

Values represent adjusted estimates from GEE models. Among 355 hospital-years of PCU care, the mean proportion of non-cancer patients was 18.8% and was significantly higher at the secondary-level (7.3%, *p* = 0.019), community (13.5%, *p* = 0.003), and rural (20.1%, *p* < 0.001) hospitals, as well as those with more palliative care beds. In 699 hospital-years of PSC, the mean proportion of non-cancer patients was 39.7%, and it was significantly higher in hospitals without radiation oncology departments (18.1%, *p* < 0.001) and with fewer acute care beds (*p* < 0.05). In both models, the proportion of non-cancer patients steadily increased over time (*p* < 0.05).

**Conclusions:**

Metropolitan tertiary medical centers with advanced cancer-care infrastructure have not yet accommodated a sufficient number of non-cancer patients relative to their capacity for providing palliative care. Meanwhile, rural areas as well as secondary-level and community hospitals have shown potential to deliver such care to a larger number of patients. These findings suggest that institutional size, function, and geographic location all influence access to non-cancer palliative care. They also indicate a policy implication: strengthening capacity in smaller and rural hospitals may help expand system-wide service coverage for non-cancer patients at a reasonable cost.

## Background

As the global population continues to age, healthcare systems face a growing demand for high-quality palliative care beyond oncology [1–4]. While the benefits of palliative care for terminally ill cancer patients are well established [5, 6], there is an increasing recognition that individuals with non-cancer conditions, such as heart failure, chronic obstructive pulmonary disease, and dementia, experience symptom burdens and care needs that are equally profound [7].

Despite these similarities, non-cancer patients are consistently underrepresented in palliative care services worldwide. A 10-year review in the United Kingdom (UK) revealed that non-cancer patients comprised only 3.9% of palliative admissions from 1993 to 1997, increasing modestly to 7.7% between 2008 and 2012 [8]. In Germany, data from 2007 to 2011 showed that only 8.1% of non-cancer end-of-life patients received palliative care [9]. In Taiwan, from 2010 to 2015, approximately 11% of non-cancer terminal patients received palliative services, compared to 55% of cancer patients [10]. As recently as 2014 in the UK, patients with advanced non-cancer diseases were still 20% less likely to receive palliative care than those with advanced cancer (40.7% vs. 61.9%) [11]. These disparities have been attributed in part to prognostic uncertainty and complex disease trajectories typical of non-cancer illnesses, in contrast to the more predictable decline in terminal cancer [12].

Tobin et al. underscored multiple dimensions of inequality in palliative care delivery, including underrepresentation of non-cancer patients, the elderly, and those living in rural or underserved areas [13]. Similarly, Teggi et al. emphasized the prolonged health deterioration experienced by individuals over 80 years of age who died from physical disabilities, chronic, or degenerative conditions [14]. They highlighted the continuum between long-term and palliative care in late life, and the lack of policy attention, which leaves many older adults without adequate support.

In Taiwan, where palliative care is predominantly delivered through hospital-based services as opposed to hospice-based models in the United Kingdom or home-based hospice care in the United States, the structural orientation of the health system may further exacerbate disparities. Although tertiary medical centers are regarded as leaders in palliative care, they often prioritize advanced oncological treatments. By contrast, secondary-level and community hospitals, which typically operate with limited hospice resources, are more closely integrated with local and long-term care networks [15, 16].

Taiwan presents a distinctive context. As a regional leader in palliative care [17, 18], its National Health Insurance (NHI) program expanded coverage to include patients with non-cancer conditions in 2009 [19, 20]. Nevertheless, despite these policy advancements, limited research has systematically examined the care infrastructure—how hospital characteristics, institutional structures, and public health policies shape access to non-cancer palliative services. To address this gap, the present study analyzes the national distribution of palliative care services for both cancer and non-cancer patients, with the aim of identifying system-level determinants and opportunities to better unitize service provision in reasonable cost.

## Method

### Study design

Palliative care services in Taiwan include three primary modalities: inpatient palliative care units (PCUs), home-based palliative care, and palliative shared care (PSC) provided in other units. This study investigated how hospital characteristics are associated with the proportion of non-cancer palliative care provision across hospitals. This study consisted of three stages:

Data were collected at the hospital level, including the number of admissions to palliative care units (PCU model) and the number of inpatients who received their first palliative shared care in other units (PSC model). For each hospital and year, the proportion of non-cancer palliative inpatients was calculated and subsequently averaged across the five-year study period to obtain hospital-year averages.

To evaluate the associations between hospital characteristics and the proportion of non-cancer palliative inpatients, generalized estimating equations (GEE) were employed.

### Data source

Data was obtained from the NHI database maintained by the Taiwan Health and Welfare Data Science Center [21]. All data were de-identified before use. The following five datasets were utilized: (1) Inpatient expenditures by admissions (2) Inpatient detailed medical orders (3) Cause of death statistics (4) Health resources of medical facilities (5) Basic profile of NHI-contracted hospitals.

### Study groups and period

Although all types of palliative care aim to improve end-of-life quality, inpatient care is especially critical for patients with severe symptoms or insufficient family support [22]. This study focuses on hospital-based palliative care between 2016 and 2020. The inpatients were grouped into the PCU and PSC models. The study period was selected to reflect a relatively consistent policy environment. In 2015, Taiwan implemented two significant reforms: enhanced reimbursement for PCU admissions [23] and integration of the “Nine Palliative Accreditation Benchmarks” into hospital accreditation criteria [24, 25].

The year 2020 represents the most recent nationwide claims data available at the time of analysis. Taiwan’s NHI database is released with a standard one-year delay to allow for data cleaning and verification; therefore, complete hospital-level palliative care files beyond 2020 were not yet accessible when this study was conducted. Although the COVID-19 pandemic emerged in 2020, Taiwan experienced only limited community transmission during that year, and routine hospital operations, including palliative care services, remained largely unaffected. As such, the 2020 data used in this study can be considered broadly representative of pre-pandemic patterns of care.

### Dependent variables: diagnosis type and non-cancer proportion

To differentiate between cancer and non-cancer palliative patients, we cross-referenced two data fields: (1) the first three discharge diagnosis codes from the “inpatient expenditures by admissions” dataset, and (2) the death classification code from the “cause of death statistics” dataset.

Patients were categorized as cancer-related if any of these codes indicated a cancer diagnosis, regardless of whether the cancer was the immediate cause of death. For patients who were still alive at the end of 2020, the classification relied solely on the first three discharge diagnoses.

The non-cancer proportion was calculated as the number of non-cancer palliative inpatients divided by the total number of palliative inpatients per hospital-year. Table 1 summarizes all variables included in the model.

**Table 1:** List of research variables

Type	Variable	Attribute	Operational definition	Data source
Dependent variable				
	Diagnosis	CAT	Patients were classified as non-cancer patients if neither their death code nor their diagnosis codes were cancer-related. Cancer/non-cancer	IPD, CD
	Non-cancer proportion	CONTD	The proportions were calculated using “non-cancer terminal patients receiving palliative care” in different hospitals each year as the numerator and the total attendance as the denominator.	IPD, CD
Independent variable				
Time variable	Admission year	CAT	Admission year 2016/2017/2018/2019/2020	IPD
Hospital variable (both in PCU & PSC model)	Hospital level	CAT	Classification of hospital Tertiary medical center / Secondary-level hospital / Community hospital	BPCH
	Hospital region	CAT	Location of hospital Cities/suburban areas/rural areas	HRMF
	Hospital ownership	CAT	Ownership of hospital Public/ private	HRMF
	Hospital with Radiation Oncology Department	CAT	Existence of a radiation oncology department in the hospital Yes/no	HRMF
(PCU model specific)	Responsible department	CAT	Department responsible for the palliative care Family medicine/ joint family medicine and other specialties /other departments	IPD
	Palliative bed count (group)	CAT	Grouped as 1–5 / 6–10 / 11–15 /≧16 beds	HRMF
(PSC model specific)	Hospital with PCU	CAT	Existence of a PCU in the hospital providing PSC Yes/no	HRMF
	Hospital acute bed count (group)	CAT	Grouped as ≦ 200 / 201–400 / 401–600 / ≧601 beds	HRMF

(1) *CAT*  Category (2) *CONTD*  Continuous (3) *IPD*  Inpatient Expenditures by Admissions (4) *CD*  Cause of Death statistics (5) *HRMF*  Health Resources of Medical Facilities (6) *BPCH*  Basic profile of NHI-contracted hospitals

### Independent variables

Because prior research has not systematically examined hospital-level determinants of non-cancer palliative care delivery, we first reviewed all available information within the NHI database to identify hospital characteristics that were both theoretically relevant and consistently measurable across institutions. The selected variables represent structural, organizational, and resource-related factors that may reasonably influence palliative care service composition, based on clinical practice patterns and Taiwan’s healthcare system design.

To examine patterns over time and across hospital types, several hospital-level variables were included. Admission year referred to the calendar year in which the patient was admitted, ranging from 2016 to 2020. Hospital level was categorized according to Taiwan’s three-tier accreditation system: tertiary medical centers, which are comprehensive facilities involved in teaching, research, and advanced care; secondary-level hospitals, which are regional general hospitals offering specialist services; and community hospitals, which are smaller district-level institutions with limited specialization. Two hospitals were reclassified to better reflect their roles in palliative care delivery. Hospital region was grouped into three categories based on geographic location: cities (municipalities in western Taiwan), suburban areas (non-municipal western regions), and rural areas (eastern Taiwan and outlying islands). Hospital ownership distinguished between public hospitals (state-run or affiliated) and private hospitals (operated by nonprofit, religious, or private organizations). The presence of a radiation oncology department was used as a proxy indicator for the availability of advanced cancer treatment infrastructure.

For the PCU model, additional variables included the department responsible for leading the PCU. In Taiwan, since palliative care is considered a non-departmental subspecialty, leadership typically comes from family medicine or oncology-related departments. These were grouped into three categories: family physicians alone, joint leadership by family physicians and other specialties, and other departments. The number of palliative care beds per hospital, often limited due to staffing constraints, was categorized into 1–5, 6–10, 11–15, and ≥ 16 beds.

For the PSC model, variables included whether the hospital had a formal PCU, which in Taiwan requires compliance with standards set by the Taiwan Academy of Hospice Palliative Medicine. Due to limited resources, many hospitals only offer PSC. Hospital size was evaluated using the number of acute care beds, categorized as ≤ 200, 201–400, 401–600, and ≥ 601 beds, to explore its association with the proportion of non-cancer palliative care cases.

### Data filtering and integration

**Fig. 1:**
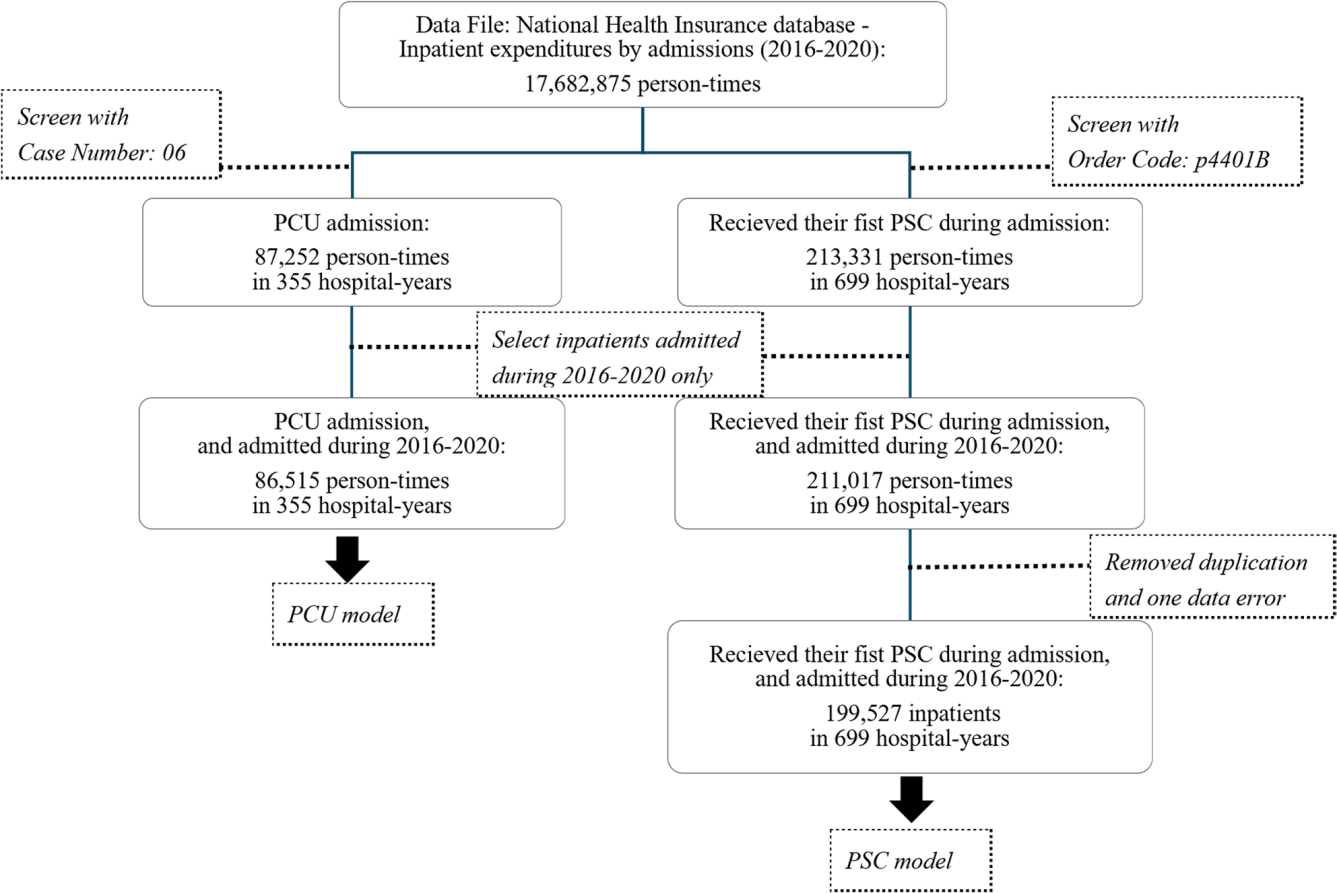
Screening flow chart

Data filtering and integration were performed using SAS version 9.4 [26]. Admissions dated from January 1, 2016, to December 31, 2020, were included in the analysis. One patient with inconsistent records (death before admission) was excluded. For the PCU model, the main file “Inpatient Expenditures by Admissions” was filtered using case type code “06,” indicating a PCU admission. For the PSC model, the file “Details of Inpatient Orders” was filtered using order code “P4401B,” indicating receipt of a first PSC consultation during that admission. Duplicate entries, due to multiple admissions to different hospitals or repeated declarations, were removed. (Fig. 1)

The final analysis was performed using STATA version 15 [27]. Prior to multivariable modeling, univariable comparisons were conducted to examine differences in the proportion of non-cancer patients across hospital characteristics. Specifically, *p* values in Tables 2 and 3 were derived from one-way ANOVA for multi-group comparisons and independent-samples *t* tests for two-group comparisons.

**Table 2:** Hospital-year count and average non-cancer proportion for PCU model

Variable Item		Total (hospital-year)	Mean(SD) of non-cancer proportion	
				*p* value
Total		355	18.8% (0.161)	
Number of palliative beds^**^	1–5 beds	40	15.1% (0.186)	0.006
	6–10 beds	139	16.9% (0.172)	
	11–15 beds	93	24.5% (0.168)	
	≧ 16 beds	83	17.3% (0.091)	
Hospital level^***^	Tertiary medical centers	90	13.7% (0.086)	< 0.001
	Secondary-level hospitals	197	18.2% (0.137)	
	Community hospitals	68	27.1% (0.247)	
Hospital region^***^	Cities	208	14.9% (0.092)	< 0.001
	Suburban areas	105	18.9% (0.189)	
	Rural areas	42	37.4% (0.216)	
Hospital ownership	Public	149	20.7% (0.205)	0.067
	Private	206	17.3% (0.118)	
Departments responsible for PCU^**^	Family medicine	146	22.0% (0.188)	0.002
	Joint family medicine and others	41	25.1% (0.165)	
	Other departments	168	14.4% (0.117)	
Hospitals with a radiation oncology department^**^	No	92	24.6% (0.225)	0.001
	Yes	263	16.7% (0.125)	
Admission year^**^	2016	62	15.6% (0.165)	0.004
	2017	66	16.4% (0.131)	
	2018	71	17.7% (0.158)	
	2019	77	20.8% (0.164)	
	2020	79	22.2% (0.173)	

*p* < 0.05^*^,*p* < 0.01^**^, *p* < 0.001^***^

**Table 3:** Table 3 Hospital-year count and average non-cancer proportion for PSC model

Variable Item			Total (hospital-year)	Mean(SD) of non-cancer proportion	*p* value
Total			699	39.7% (0.250)	
Number of hospital acute bed^***^		≦ 200 beds	215	54.4% (0.299)	< 0.001
		201–400 beds	230	38.2% (0.235)	
		401–600 beds	111	29.9% (0.156)	
		≧ 601 beds	143	27.7% (0.102)	
Hospital level^***^		Tertiary medical Center	95	27.8% (0.090)	< 0.001
		Secondary-level Hospital	350	34.6% (0.207)	
		Community Hospital	254	51.2% (0.298)	
Hospital region		Cities	389	37.3% (0.233)	0.056
		Suburban areas	228	41.4% (0.271)	
		Rural areas	82	46.2% (0.256)	
Hospital ownership		Public	251	41.9% (0.241)	0.09
		Private	448	38.5% (0.255)	
Hospitals with a PCU^***^		No	341	44.5% (0.277)	< 0.001
		Yes	358	35.1% (0.212)	
Hospitals with a radiation oncology department^***^		No	294	53.0% (0.288)	< 0.001
		Yes	405	30.0% (0.161)	
Admission year^**^		2016	133	34.2% (0.269)	0.001
		2017	134	37.2% (0.247)	
		2018	142	38.3% (0.249)	
		2019	145	43.5% (0.238)	
		2020	145	44.7% (0.236)	

Note: *p* < 0.05^*^ 、*p* < 0.01^**^、 *p* < 0.001^***^

GEE were applied to stratify data by admission year and hospital characteristics. Although multiple hospital characteristics may simultaneously influence the proportion of non-cancer to cancer patients, these factors often overlap and are interrelated. Furthermore, previous studies have demonstrated a gradual annual increase in the proportion of non-cancer patients [8–11]. GEE was selected to allow stratification by time while more precisely assessing the independent effects of hospital characteristics on the non-cancer proportion, thereby improving the accuracy of estimating the association between hospital characteristics and the delivery of non-cancer palliative care. These models accounted for the bounded nature of the outcome variable (ranging from 0 to 1).

## Results

### Descriptive overview and univariable analysis of the PCU model

Between 2016 and 2020, 86,515 PCU admissions were recorded, including 69,681 cancer patients and 16,834 non-cancer patients. The number of hospitals operating PCUs increased from 62 in 2016 to 79 in 2020, and a total of 355 hospital-years. The proportion of non-cancer patients had steadily increased over time (Fig. 1).

In the univariable analyses (Table 2), the proportion of non-cancer PCU admissions varied significantly across hospital characteristics. Community hospitals and rural hospitals had the highest non-cancer proportions, while tertiary centers and city hospitals reported the lowest (*p* < 0.001). Higher proportions were also observed in hospitals with family medicine involvement (*p* = 0.002) and in those without radiation oncology services (*p* = 0.001). A temporal increase was noted, rising from 15.6% in 2016 to 22.2% in 2020 (*p* = 0.004).

### Descriptive overview and univariable analysis of the PSC model

From 2016 to 2020, 199,527 inpatients received their first PSC during hospitalization, including 134,973 cancer patients and 64,554 non-cancer patients. The number of hospitals offering PSC rose from 133 in 2016 to 145 in 2020 and a total of 699 hospital-years. Similar to the PCU model, the proportion of non-cancer patients increased annually (Fig. 1).

In the univariable analyses (Table 3), the proportion of non-cancer PSC patients varied significantly across hospital characteristics. Smaller hospitals (≤ 200 beds) and community hospitals consistently showed higher non-cancer proportions compared with larger and tertiary (*p* < 0.001). Hospitals without PCUs or radiation oncology departments also had higher non-cancer representation (both *p* < 0.001). A clear temporal increase was observed, rising from 34.2% in 2016 to 44.7% in 2020 (*p* = 0.001).

### Non-cancer proportion of PCU model analyzed with GEE

**Fig. 2:**
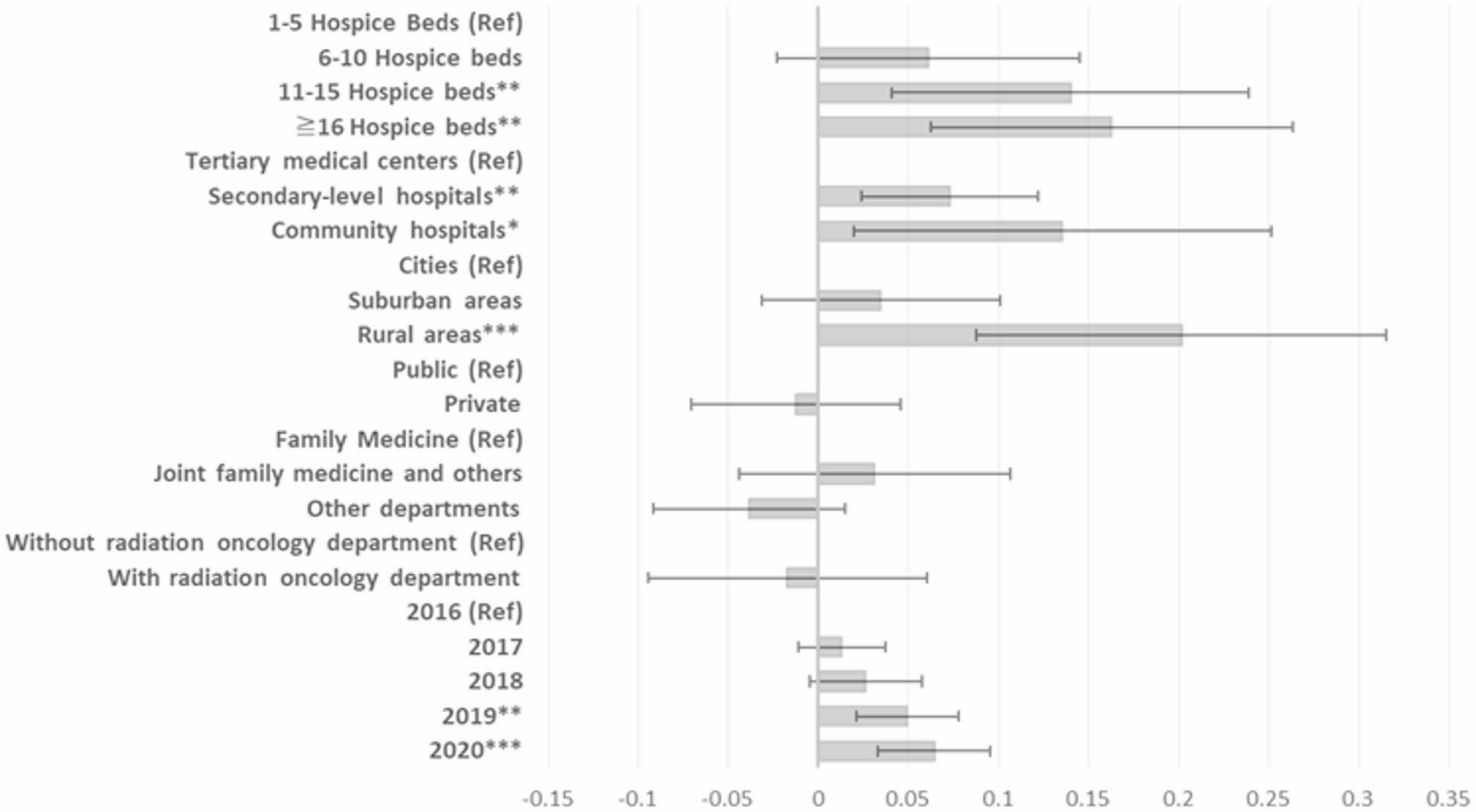
Bar chart with confidence intervals for GEE analysis: non-cancer proportion of PCU model

Table 4; Fig. 2 summarize the GEE analysis of non-cancer proportions in the PCU model. Compared with 2016, the non-cancer proportion increased by 1.3% in 2017 (*p* = 0.281) and 2.6% in 2018 (*p* = 0.090), although these differences were not statistically significant. However, the increases in 2019 (4.9%, *p* = 0.001) and 2020 (6.4%, *p* < 0.001) were statistically significant.

**Table 4:** GEE analysis of the non-cancer proportion in PCU compared to benchmarks

Variable Item			Coefficient	Standard error	Z	*p* > |z|	95% Confidence interval	
Number of palliative bed	Benchmark: 1–5 beds							
	6–10 beds		0.061	0.042	1.45	0.146	-0.021	0.143
	11–15 beds**		0.140	0.050	2.82	0.005	0.042	0.237
	≧ 16 beds**		0.163	0.050	3.25	0.001	0.065	0.261
Hospital level	Benchmark: Tertiary medical centers							
	Secondary-level hospitals**		0.073	0.024	2.99	0.003	0.025	0.121
	Community hospitals*		0.135	0.058	2.34	0.019	0.022	0.249
Hospital region	Benchmark: Cities							
	Suburban areas		0.035	0.033	1.04	0.296	-0.030	0.100
	Rural areas***		0.201	0.057	3.55	< 0.001	0.090	0.313
Hospital ownership	Benchmark: Public							
	Private		-0.012	0.029	-0.43	0.67	-0.069	0.044
Responsible department	Benchmark: Family medicine							
	Joint family medicine and others		0.031	0.038	0.82	0.41	-0.043	0.105
	Other departments		-0.039	0.026	-1.46	0.146	-0.090	0.013
Hospitals with a radiation oncology department	Benchmark: No							
	Yes		-0.017	0.039	-0.45	0.655	-0.093	0.058
Admission year	Benchmark: 2016							
	2017		0.013	0.012	1.08	0.281	-0.011	0.036
	2018		0.026	0.016	1.69	0.09	-0.004	0.057
	2019**		0.049	0.014	3.46	0.001	0.021	0.077
	2020***		0.064	0.016	4.13	< 0.001	0.034	0.095

*p* < 0.05^*^, *p* < 0.01^**^, *p* < 0.001^***^

Regarding palliative bed capacity, hospitals with 6–10 beds did not differ significantly from the reference group (1–5 beds) (*p* = 0.146). However, hospitals with 11–15 beds and ≥ 16 beds showed significantly higher non-cancer proportions, by 14.0% (*p* = 0.005) and 16.3% (*p* = 0.001), respectively.

When stratified by hospital level, secondary-level hospitals had a 7.3% higher non-cancer proportion (*p* = 0.003) and community hospitals had a 13.5% higher proportion (*p* = 0.019) than tertiary medical centers.

Geographically, rural hospitals had a significantly higher non-cancer proportion than cities’, with a 20.1% increase (*p* < 0.001). No significant difference was observed between suburban and urban hospitals (*p* = 0.296).

### Non-cancer proportion for PSC model analyzed with GEE

**Fig. 3:**
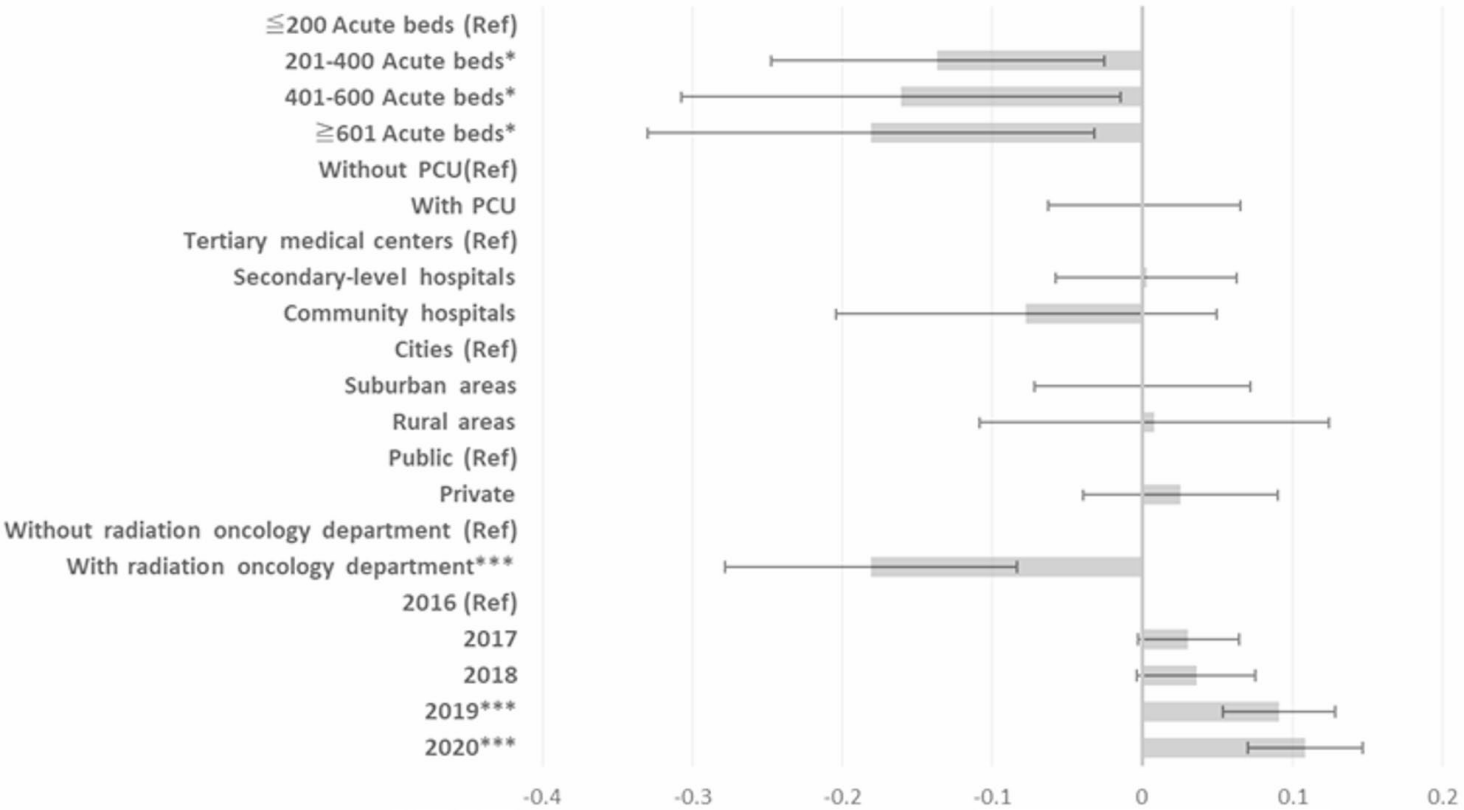
Bar chart with confidence intervals for GEE analysis: non-cancer proportion for PSC model

Table 5 and Fig. 3 present the GEE results of the PSC model. Compared with 2016, the non-cancer proportion increased by 3.0% in 2017 (*p* = 0.070) and 3.6% in 2018 (*p* = 0.071), although these changes were not statistically significant. However, there was a significant increase of 9.1% in 2019 (*p* < 0.001) and 10.9% in 2020 (*p* < 0.001).

**Table 5:** GEE analysis of the non-cancer proportion for PSC model

Variable Item		Coefficient	Standard error	z	*p* > |z|	95% Confidence interval	
Number of hospital acute beds	Benchmark: ≦200 beds						
	201–400 beds^*^	-0.137	0.056	-2.46	0.014	-0.245	-0.028
	401–600 beds^*^	-0.161	0.073	-2.2	0.028	-0.304	-0.018
	≧ 601 beds^*^	-0.181	0.074	-2.44	0.015	-0.327	-0.035
Hospitals with a PCU	Benchmark: No						
	Yes	0.001	0.032	0.04	0.966	-0.061	0.064
Hospital level	Benchmark: Tertiary medical centers						
	Secondary-level hospitals	0.003	0.030	0.09	0.931	-0.057	0.062
	Community hospitals	-0.078	0.063	-1.23	0.221	-0.202	0.047
Hospital region	Benchmark: Cities						
	Suburban areas	-0.0001	0.036	-0.01	0.996	-0.071	0.070
	Rural areas	0.008	0.058	0.13	0.897	-0.107	0.122
Hospital ownership	Benchmark: Public						
	Private	0.025	0.033	0.78	0.438	-0.039	0.089
Hospitals with a radiation oncology department	Benchmark: No						
	Yes^***^	-0.181	0.049	-3.72	< 0.001	-0.276	-0.085
Admission year	Benchmark: 2016						
	2017	0.030	0.017	1.81	0.07	-0.002	0.063
	2018	0.036	0.020	1.81	0.071	-0.003	0.075
	2019^***^	0.091	0.019	4.88	< 0.001	0.054	0.127
	2020^***^	0.109	0.019	5.71	< 0.001	0.071	0.146

*p* < 0.05^*^ ,  *p* < 0.01^**^,  *p* < 0.001^***^

Hospitals without radiation oncology departments had a significantly higher proportion of non-cancer PSC admissions. Conversely, those with such departments showed an 18.1% reduction in non-cancer PSC admissions (*p* < 0.001).

With respect to hospital size, institutions with 201–400, 401–600, and ≥ 601 acute care beds had significantly lower non-cancer proportions than those with ≤ 200 beds. Specifically, the proportions were reduced by 13.7% (*p* = 0.014), 16.1% (*p* = 0.028), and 18.1% (*p* = 0.015), respectively.

## Discussion

This study identifies key hospital-level factors associated with the provision of non-cancer palliative care across Taiwan’s dual inpatient care models: PCU and PSC. Because palliative care services are fully reimbursed under Taiwan’s NHI, all hospitals providing PCU or PSC services are captured in the claims database. Thus, the reported proportions represent nationwide values among hospitals that provide these services. The findings provide important insights into how institutional characteristics shape patterns of service utilization and may offer relevant considerations for other health systems with hospital-based palliative care structures.

The steady increase in non-cancer palliative proportions from 2016 to 2020, particularly within the PSC model, signals growing system responsiveness to non-cancer populations. PSC services are delivered across multiple departments and care settings, enabling broader access to patients with unpredictable trajectories and diagnoses outside traditional cancer-centric frameworks [28]. This flexibility may support broader inclusion of non-cancer populations and offers insights for health systems that rely on hospital-based palliative care models.

Hospitals with greater palliative bed capacity demonstrated significantly higher non-cancer proportions, suggesting that infrastructure influences the ability to accommodate patients with diverse needs. Smaller units, which often operate with more limited staffing and resources, may serve proportionally more cancer patients whose disease courses are more predictable. These findings indicate that strengthening capacity where feasible may help broaden access for patients with conditions such as frailty, organ failure, or dementia.

Our results also showed that non-cancer palliative admissions were more common in secondary-level and community hospitals than in tertiary medical centers. This discrepancy may relate to the concentration of high-intensity oncologic services in tertiary centers, resulting in a different case composition. By contrast, smaller hospitals, particularly those in rural areas, are often more integrated with long-term care facilities and community-based networks, which may increase the likelihood of encountering non-cancer patients [15, 16]. As the population continues to age, particularly in rural areas, enhancing capacity across multiple levels of the health system may support more balanced and sustainable palliative care provision.

Additionally, hospitals without radiation oncology departments and with fewer acute care beds reported significantly higher non-cancer proportions in the PSC model. In large hospitals, patterns of PSC utilization may be influenced by competing demands from cancer-focused services, which constitute a substantial component of palliative care activity. These findings suggest that service orientation and resource allocation at the institutional level may shape the relative inclusion of non-cancer patients across different hospital types.

These differences may partly reflect the structural functions and policy orientations of Taiwan’s healthcare system rather than inequities per se. As the population continues to age, cancer treatments diversify, and the number of non-cancer palliative care cases rises, expanding service capacity beyond oncology may become increasingly relevant for meeting evolving needs. Taken together, these findings suggest that enhancing capacity across rural, secondary-level, and community hospitals could support broader and more sustainable system-wide coverage. Such efforts may contribute to a more balanced palliative care network that aligns with demographic trends and improves access for patients with non-cancer, life-limiting conditions.

### Study limitation

This study had several limitations. First, although the analysis was based on comprehensive nationwide data from Taiwan National Health Insurance system, the findings may not be directly generalizable to countries with different healthcare financing or delivery models. Nonetheless, the identified challenges, such as the underrepresentation of non-cancer patients and the distinct roles of tertiary versus community-level hospitals, may be relevant across aging societies worldwide.

Second, while hospital-level factors were examined, we did not include patient-level clinical data, such as specific diagnoses, functional status, or symptom burden. These factors could enhance the understanding of utilization patterns but were beyond the scope of this structural analysis. However, the institutional-level focus provides meaningful insight into system-level influences on access to palliative care.

Third, although the study distinguished between PCU and PSC delivery models, the data did not capture nuances, such as care quality, decision-making processes, or interdisciplinary team composition. Future research should incorporate qualitative methods or patient-reported outcomes to further explore care experiences among non-cancer populations.

Despite these limitations, this study offers a system-level perspective on palliative care provision within a mature, universal health system. These findings may be informative for health systems seeking to broaden access to palliative care for patients with non-cancer conditions.

## Conclusion

This study examined how hospital characteristics are associated with access to non-cancer palliative care within the universal health system. Tertiary medical centers with advanced oncologic capabilities have not yet accommodated a sufficient number of non-cancer patients relative to their capacity for providing palliative care, whereas rural, secondary-level, and community hospitals have shown potential to deliver such care to a larger number of patients.

Our findings indicated that hospital size, geographic location, and organizational structure were associated with variations in the inclusion of non-cancer patients in palliative care services. These patterns are consistent with the broader systemic trends observed in aging societies and may be informative for health systems seeking to support palliative care delivery across diverse care settings.

Strengthening palliative care capacity in non-tertiary hospitals, particularly those in rural or less-resourced regions, may help broaden access for patients with non-cancer, life-limiting conditions. Future research should explore the applicability of these findings across different healthcare models and identify strategies to enhance the integration of non-cancer palliative services.

## Data Availability

The data that support the findings of this study are available from the National Health Insurance Research Database maintained by the Taiwan Health and Welfare Data Science Center. Due to legal and ethical restrictions, these data are not publicly available. Access is limited to on-site use at designated subcenters authorized by the Ministry of Health and Welfare. Data are available from the authors upon reasonable request and with permission from the Data Science Center.

## Abbreviations

PCU: Palliative care unit
PSC: Palliative shared care
NHI: National health insurance
GEE: Generalized estimating equations
